# Zero budget natural farming components Jeevamrit and Beejamrit augment *Spinacia oleracea* L. (spinach) growth by ameliorating the negative impacts of the salt and drought stress

**DOI:** 10.3389/fmicb.2024.1326390

**Published:** 2024-03-12

**Authors:** Margi Patel, Shaikhul Islam, Bernard R. Glick, Nisha Choudhary, Virendra Kumar Yadav, Snehal Bagatharia, Dipak Kumar Sahoo, Ashish Patel

**Affiliations:** ^1^Department of Life Sciences, Hemchandracharya North Gujarat University, Patan, Gujarat, India; ^2^Plant Pathology Division, Bangladesh Wheat and Maize Research Institute, Dinajpur, Bangladesh; ^3^Department of Biology, University of Waterloo, Waterloo, ON, Canada; ^4^Gujarat State Biotechnology Mission, Gandhinagar, Gujarat, India; ^5^Department of Veterinary Clinical Sciences, College of Veterinary Medicine, Iowa State University, Ames, IA, United States

**Keywords:** abiotic stresses, plant growth promoting bacteria, sustainable agriculture, traditional biofertilizer, zero budget natural farming (ZBNF)

## Abstract

The growth of crop plants, particularly spinach (*Spinacia oleracea* L.), can be significantly impeded by salinity and drought. However, pre-treating spinach plants with traditional biofertilizers like Jeevamrit and Beejamrit (JB) substantially reverses the salinity and drought-induced inhibitory effects. Hence, this study aims to elucidate the underlying mechanisms that govern the efficacy of traditional fertilizers. The present work employed comprehensive biochemical, physiological, and molecular approaches to investigate the processes by which JB alleviates abiotic stress. The JB treatment effectively boosts spinach growth by increasing nutrient uptake and antioxidant enzyme activity, which mitigates the detrimental effects of drought and salinity-induced stress. Under salt and drought stress conditions, the application of JB resulted in an impressive rise in germination percentages of 80 and 60%, respectively. In addition, the application of JB treatment resulted in a 50% decrease in electrolyte leakage and a 75% rise in the relative water content of the spinach plants. Furthermore, the significant reduction in proline and glycine betaine levels in plants treated with JB provides additional evidence of the treatment's ability to prevent cell death caused by environmental stressors. Following JB treatment, the spinach plants exhibited substantially higher total chlorophyll content was also observed. Additionally, using 16S rRNA sequencing, we discovered and characterized five plant-beneficial bacteria from the JB bio-inoculants. These bacterial isolates comprise a number of traits that contribute to growth augmentation in plants. These evidences suggest that the presence of the aforesaid microorganisms (along with additional ones) is accountable for the JB-mediated stimulation of plant growth and development.

## 1 Introduction

Abiotic stresses are significant limitations that have detrimental effects on the physical and physiological characteristics of plants. These stresses also prevent plants from carrying out their normal functions and ultimately cause a considerable decrease in agricultural output (Soni et al., 2023). The co-existence of several abiotic stressors, instead of one particular stress condition, can induce an array of biochemical and physiological events, resulting constrained development, impaired metabolism, and restricted growth (Shabbir et al., 2022). Amongst the abiotic stress factors, salinity and drought are the main global concerns that are a severe threat to vegetable productivity (Hossain et al., 2022; Khalid et al., 2023). Plants like vegetable crops are typically able to survive under diverse environmental stress situations as a consequence of natural adaptation and harmonization mechanisms, however these capabilities are not always adequate to fight against abrupt environmental fluctuations (Dhankher and Foyer, 2018). Salinity and drought significantly affect the vegetable production. Most vegetables exhibit susceptibility to salinity, with a critical threshold at an electrical conductivity (EC) of ~2.5 dS m^−1^ (Behera et al., 2022). Moreover, these plants exhibit a heightened susceptibility to arid circumstances when the volume of water in their bodies attains a threshold of around 20% (Parkash and Singh, 2020; Razi and Muneer, 2021).

Vegetables are known as a fundamental source of the human diet due to the fact that they contain an abundance of vitamins, minerals, dietary fibers, and antioxidants. The risk of several illnesses can be decreased by the dietary intake of an appropriate amount of certain vegetables (Altemimi et al., 2015; Gamalero and Glick, 2022). Spinach has been proven to be a vital source of dietary fibers, beta-carotene, ascorbic acid, phosphorus, calcium, potassium, iron, and bioactive components like polyphenols, glucuronic acid, and 20-hydroxyecdysone. Furthermore, it is abundant source of many essential minerals and vitamins, particularly vitamin C, which confers advantageous effects on human wellbeing (Ekinci et al., 2015; Xu and Leskovar, 2015; Massa et al., 2018; Bokov et al., 2020). Throughout the dietary manufacturing sector, consumer interest in the spinach continues to rise substantially (Nguyen et al., 2019). The primary factors limiting its growth are drought and salt stresses, which have an impact on the nutritional value and stimulate physiological alterations in spinach (Zaghdoud et al., 2012; Wu et al., 2013; Sun et al., 2023).

The issues of food production and environmental sustainability are among the foremost challenges that humanity faces in the current day. Conventional farming practices are widely recognized as unsustainable and inadequate in addressing significant societal challenges such as environmental degradation, food safety, climate change, and reliance on non-renewable energy sources (Rosati et al., 2021). The Green Revolution was successful in increasing food production but at the expense of environmental damage confronting a deep crisis (IPES-Food, 2016). Therefore, researchers recommend using environmentally friendly farming methods to increase vegetable yield in salinity and drought-stressed environments. The usage of cover crops and the application of manure might be the alternative farming practices that have the greatest effect on enhancing agricultural sustainability (Teasdale et al., 2007; Muller and Aubert, 2014).

The task of providing sustenance for an expanding global population, anticipated to reach between 9 and 10 billion individuals by the year 2050, presents a formidable challenge. As a result, globally adopting sustainable agricultural methods is an excellent way to address this major issue and secure the future safety of both the environment and food [UNEP (United Nations Environment Programme), 2009; The National Academies, 2010; United Nations Digital Library, 2011]. Traditional farming techniques have been a crucial component of India's food production since prehistoric times. These techniques have the capability to alleviate the adverse consequences of changing climate through the utilization of several geographical and temporal approaches. Conversely, the monoculture and the improper consumption of fossil fuels and agrochemicals have led to a deterioration of the socio-ecological impact on the environmental conditions (Phungpracha et al., 2016). Conventional agriculture's ability to adapt to and mitigate these anthropogenic hazards can help to ensure long-term production and environmental security (Watson, 2019). It uses location-specific farming methods that are suitable for specific environmental variables, spatial diversity, and prudent use of the available local resources to maintain productivity (Lincoln, 2019).

The archaic and decades-old farming technique recognized as zero-budget natural farming (ZBNF) has recently gained popularity as a self-sufficient solution that could assist in solving the aforementioned concerns. Thousands of farmers in India have employed ZBNF, which uses natural resources, numerous cropping methods, and products based on urine and cow dung to enhance soil biology. Since there are no credit requirements in ZBNF, the cost of cultivation operations stays zero, allowing crops to be grown without chemicals by utilizing naturally occurring resources (Timsina, 2018; Bharucha et al., 2020; Bhattacharyya et al., 2020; Khadse and Rosset, 2021). Beejamrit, Panchgavya, and Jeevamrit are examples of ZBNF formulations that promote microbial life and earthworm activities, which promote soil nutrient availability, affirm disease resistance, and improve the productivity of crops (Maduka and Udensi, 2019; Ray et al., 2020; Patel et al., 2021). Beejamrit and Jeevamrit are recognized as pillars of ZBNF (Bharadwaj, 2021). Beejamrit, an indigenous biofertilizer used to treat seeds, consists primarily of cow dung and urine, both of which are rich in bacteria, fungus, and actinomycetes (Swaminathan, 2005). Jeevamrit is composed of cow urine, jaggery, cow dung, pulse flour, and a trace of soil, which helps to promote a healthy soil microbiome (Boraiah et al., 2017). Jeevamrit and Beejamrit (JB) application in the soil has the efficiency to improve soil fertility and plant development. These practices should be promoted and implemented, especially by small and marginal farmers, as they lower the cost of agrochemicals while boosting the Benefit-Cost-Ratio (BCR). Despite being a traditional and longstanding method of agriculture, Zero Budget Natural Farming (ZBNF) has not received significant recognition from the scientific world, leading to uncertainty in its acceptance. This hesitancy stems from the absence of substantial scientific evidence that substantiates its effectiveness (Saharan et al., 2023). Hence our study intended to identify the fundamental mechanisms that regulate JB-mediated spinach growth enhancement after salinity and drought exposure.

## 2 Materials and methods

### 2.1 Jeevamrit and Beejamrit formulation

Jeevamrit and Beejamrit were formulated by mixing the ingredients in a plastic container (Table 1).

**Table 1:** Formulation of Jeevamrit and Beejamrit.

**Jeevamrit (1 L)**	**Beejamrit (1 L)**
Fresh cow dung—55 g	Fresh cow dung—250 g
Cow urine—55 ml	Cow urine—250 ml
Organic jaggery—11 g	Lime (calcium carbonate)—2.5 g
Chickpea flour—11 g	Farm soil—3 g
Farm soil—3 g	Water—1 L

The mixture was stirred regularly three times a day with a wooden stick. The container was covered with damp gunny sacks and kept under shade to facilitate the fermentation process. The mixture was fermented for 7 days and 24 h for Jeevamrit and Beejamrit, respectively.

### 2.2 Experimental setup and pot study

For the pot experiment, spinach seeds (minimum 65% germination rate, 98% genetic purity, and 98% physical purity) were surface sanitized using ethanol (70%) for 1 min and sodium hypochlorite (0.5%) for 5 min, then five times cleaned using sterile distilled water (SDW). Pot tests were carried out using a completely randomized block design to see whether JB can reduce salt and drought stress in spinach plants. Three growth conditions were chosen for JB and non-JB treatments: control (non-stressed), salt stress (4 and 6 dS m^−1^), and drought stress (moderate and severe). Sowing rate was 10 seeds each pot (each treatment had 10 replications). Soil used for experiment was collected from the Hemchandracharya North Gujarat University's agricultural field (23°51′44.388″N to 72°8′3.192″E). ICAR classifies the soil as alluvial. NaCl was applied to the experimental soil to provide 4 and 6 ds m^−1^ salinity stress. The soil suspension was created with distilled water at a 1:2 soil: water ratio, and an EC meter (Elico—CM 180) evaluated its electric conductivity in dS m^−1^ to quantify and maintain the soil's salinity during pot trials.

Batool et al. (2020)'s drought stress induction approach was employed on spinach. The pots were irrigated daily with tap water at field capacity until the drought stress application phase (30 DAS, days after sowing). In severe drought, soil relative water content (SRWC) was 40% and in moderate drought, 60%. By recording soil moisture daily and accounting for water loss, these water deficit circumstances lasted 7 days. However, the control pots were well-hydrated at 80% SRWC. After 7 days of water shortage, all pots were re-watered until crops matured (45 DAS). The soil's water state was assessed before adding water to plant pots by employing the following formula:


$$\begin{array}{l} SRWC\;\left(\%\right)=\left(FW-\frac{DW}{TW}\right)-\left(DW\times 100\right) \end{array}$$

The pot test soil was prepared. First, dried it naturally in the air and sieved using a 2 mm mesh to get rid of coarse particles. Next, an autoclave sterilized the soil. Finally, 5 kg of prepared soil was carefully placed in a 10 cm-high, 25 cm-wide plastic bag. Surface-sterilized spinach seeds were submerged in Beejamrit for 12 h, air-dried, and sown. Jeevamrit pre-soaking of the soil was done before planting. Each pot received 10 seedlings placed 2–2.5 cm deep. Foliar sprays of 20% Jeevamrit diluted with water were applied every 10 days. To establish control conditions, seeds were drenched in SDW, and plants were hydrated entirely with it. The experiment was done in an open greenhouse with ambient temperature and lighting.

### 2.3 Estimation of physiological and biochemical growth parameters of spinach

Physiological attributes such as plant's shoot height (cm) was measured from tip of plant to the end of stem by utilizing a scale. Root length (cm) was determined from collar area to the root end by utilizing a scale. Fresh weight (g) of shoot and root was estimated using weighing machine directly after harvest. Dry weight (g) of shoot and root was calculated using weighing balance after dehydrating in hot air oven for 4 at 40°C when constant weight was achieved. The leaf area (cm^2^) was measured by multiplying the leaf width (cm) by the leaf length (cm). Seed germination percentage was noted on 10 days after sowing (DAS), and percent (%) seed germination (Patel et al., 2023a) was determined using the below mentioned formula:


$$\begin{array}{l} \text{Seed germination }\left(\text{\%}\right)=\frac{\text{No}.\text{ of seeds germinated}}{\text{Total No}.\text{of seeds sown}}\;\times \;100 \end{array}$$

Biochemical analysis includes total chlorophyll, total soluble sugar, proline, total free amino acids, glycine betaine, lipid peroxidation, membrane permeability, relative water content, antioxidant enzymes and both macro- and micro-nutrients. The total chlorophyll amount was assessed by following the protocol described by Arnon (1949). Approximately 1 g of plant leaves were taken and homogenized in a pre-chilled mortar and pestle with 80% (V/V) acetone and a pinch of CaCO_3_. The extract was put in centrifuge for 15 min at 3,834 × g and made up to 25 ml using 80% (V/V) acetone. The optical density (OD) of clear suspension was measured at 645 nm and 663 nm in spectrophotometer. The total chlorophyll amount was quantified as μg chlorophyll per gram fresh weight of the plant leaves. The content of chlorophyll “a” and “b” were calculated using the following formula:


$$\begin{array}{l} \text{ Chlorophyll }“\text{a” }(\text{ug/ml})=\;\left(12.7\;\times \text{OD at }663\text{ nm}\right)\\ \;-\;(2.69\;\times \text{OD at }645\text{ nm})\;\\ \text{ Chlorophyll }“\text{b” }(\text{ug/ml})=\;\left(22.9\;\times \text{OD at }645\text{ nm}\right)\\ \;-\;(4.08\;\times \text{OD at }663\text{ nm})\\ \text{ Total chlorophyll }(\text{ug/ml})=\;\left(20.2\;\times \text{OD at }645\text{ nm}\right)\\ \;+(8.02\;\times \text{OD at }663\text{ nm})\; \end{array}$$

The phenol sulfuric acid procedure was used to calculate the total soluble sugar amount present in the spinach leaves. The content of total sugar was estimated using the glucose standard curve (Krishnaveni et al., 1984). Proline amount was determined using protocol defined by Patel et al. (2014). Proline was obtained by grinding 100 mg of spinach leaves in 3% sulfosalicylic acid (2 ml). Then the mixture was put in centrifuge for 10 min at 10,000 × g. The OD was recorded at 520 nm and amount of proline was measured from a standard curve by employing the below mentioned formula:


$$\begin{array}{l} \left(\text{ug proline in extract}/111.5\right)\;/\text{ g of sample}\\ =\;{\text{umol g}}^{-1}\text{ of fresh tissue} \end{array}$$

Quaternary ammonium compounds were obtained from plant tissue sample and estimated as glycine betaine equivalents by using the procedure proposed by Grieve and Grattan (1983). The formation rate of malondialdehyde (MDA) was measured to estimate the lipid peroxidation. About 1 g of spinach leaves were crushed in 10 ml trichloroacetic acid (10%) and the crushed mixture was put in centrifuge at 42,600 × g for 20 min. The reaction solution (2 ml thiobarbituric acid and 2 ml sample extract) was heated for 30 min at 95°C, immediately chilled on ice cubes, and then centrifuged at 42,600 × g for 20 min. The OD of the supernatant was evaluated at 532 nm (A532), 450 nm (A450), and 600 nm (A600). The malondialdehyde content was calculated using the following equation:


$$\begin{array}{l} \text{MDA content}=6.45\;\left(\text{A}532\;-\text{A}600\right)\;-0.56\text{ A}450 \end{array}$$

The measurement of total free amino acids was carried out according to Sadasivam and Manickam (1996). An aliquot of spinach tissue extract was made up to 1 ml using distilled water and then 1 ml ninhydrin reagent was added. The mixture was kept in a boiling water bath for 20 min. Later the mixture was chilled, and 5 ml of diluents added to each test tube. The OD was recorded at a wavelength of 570 nm. Electrolyte leakage rate (ELR) was estimated as proposed by Lutts et al. (1996) with few changes. Approximately 100 mg of fresh plant leaves were chopped into small parts and placed into a boiling test tube having 20 ml deionized water and then the electrical conductivity (EC_1_) of the bathing suspension was measured. The tubes were incubated for 3 h at 30°C and the EC_2_ was recorded. Subsequently, the sample tubes were subjected to autoclave for 20 min at 121°C to liberate all the electrolytes and lastly allowed to cool, and then the EC_3_ was estimated. The ELR was estimated using following equation:


$$\begin{array}{l} \text{ELR}\left(\text{\%}\right)=\left(\text{EC}2-\text{EC}1/\text{EC}3\right)\times 100 \end{array}$$

The relative water content (RWC) percentage (%) was assessed by following the procedure described by Teulat et al. (2003). Well-developed spinach leaves were taken and Fresh weight of was noted. Then, plant leaves were dipped in a test tube containing distilled water and kept in a refrigerator to incubate for 24 h. Afterwards, the leaves were blotted dry with the help of tissue paper and the fully turgid weight was recorded. The leaves were dehydrated in oven for 24 h at 72°C. Finally, the dry weight was estimated and the RWC was calculated using following equation:


$$\begin{array}{l} \text{RWC }\left(\text{\%}\right)=\frac{\text{Fresh weight }-\text{ Dry weight}}{\text{Fully turgid weight }-\text{ Dry weight}}\times 100 \end{array}$$

Fresh spinach leaves (0.5 g) were grinded in 5 ml of 0.2 M chilled potassium phosphate buffer (pH 7.8 with 0.1mM EDTA) to extract enzymatic antioxidants. The homogenate solution was put in a centrifuge at 4°C for 20 min at 10,000 × g. Then, extract was preserved at−20°C and utilized within 2 days to evaluate various enzymatic antioxidant activity. Superoxide dismutase, glutathione reductase, catalase, and ascorbate peroxidase were assessed according to Nakano and Asada (1981), Aebi and Lester (1984), Beyer and Fridovich (1987), and Smith et al. (1988), respectively.

Micro- and macronutrient amount present in spinach was evaluated by employing the protocol mentioned by Vaghela et al. (2010). The spinach leaves were crushed using mortar and pestle. The Kjeldahl and chlorostannous molybdophosphoric blue color techniques were followed to quantify the total nitrogen and phosphorus content, respectively. After performing triacid (HNO_3_:H_2_SO_4_:HClO_4_ in the ratio of 10:1:4) digestion, Zn, Mg, Ca, Fe, K, Mn, and Cu concentrations were estimated with the help of atomic absorption spectroscopy.

### 2.4 Assessment of physicochemical and microbial characteristics of pot experimental soil

The experimental pot soil was investigated twice (before and after JB treatment) for their physicochemical properties in accordance with the methods stated previously (Patel et al., 2023a). The standard serial dilution spread plate method was implemented to partially assess the dominant and viable pot soil microbiome where the microbiome population was quantified as colony forming unit per gram of soil (cfu g^−1^ soil) (Rao, 2005).

### 2.5 Isolation and identification of PGPB from Jeevamrit and Beejamrit

Solutions prepared using JB were diluted (10^−1^-10^−9^), and the final three dilutions were distributed across different medium (such as—Pikovskya's medium, Luria Bertani agar, Nutrient agar, Tryptone soya agar and Jensen's medium) in amounts of 0.1 ml each. The petri plates were incubated at 28 ± 2°C and observed for the presence of colonies for a maximum duration of 7 days. Repetitive sub-culturing was performed in order to obtain pure bacterial cultures.

The chosen bacteria were observed for their morphological characters. Gram staining and biochemical tests were done according to Cappuccino and Sherman (1992). Molecular identification of isolated bacteria was done by isolating genomic DNA using the technique proposed by Chen and Kuo (1993). Subsequently, the 16S rRNA gene sequence amplification was done using the polymerase chain reaction (PCR) technique, as recommended by Weisburg et al. (1991). The phylogenetic examination was conducted using the Maximum Likelihood technique and a Tamura-Nei model, as described by Tamura and Nei (1993). Primary trees for the heuristic search were achieved directly by employing the Maximum Parsimony technique. The tree is drawn to scale, with branch lengths measured in the number of substitutions per site. Evolutionary investigations were carried out using MEGA11 (Tamura et al., 2021).

### 2.6 Assessment of plant growth-enhancing features exhibited by the isolated PGPB

#### 2.6.1 Formation of indole-3-acetic acid (IAA) and gibberellic acid (GA_3_)

The production of IAA was quantitatively assessed by introducing isolated bacterial cultures into Luria-Bertani (LB) broth that was supplemented with 2 mg ml^−1^ tryptophan, as it serves as a precursor for IAA synthesis. The cultures were then incubated at 28 ± 2°C for a period of 5 days. Afterwards, the bacterial cultures were subjected to centrifugation at a speed of 10,650 × g for a duration of 5 min. A mixture was prepared by combining equal amounts (3 ml) of supernatant and salkowski's reagent, and then few drops of orthophosphoric acid were added. The resulting mixture was then put for incubation in a dark environment for 1 h. The formation of a pink color indicates IAA production and the OD was calculated at 530 nm. A standard curve of pure IAA was utilized to measure the IAA concentration in the supernatant (Slama et al., 2019).

The isolates were introduced into Jensen's medium and subjected to incubation at a speed of 6.13 × g for a duration of 5 days at 30°C. Post incubation, supernatant was collected and separating funnel was used to filter the supernatant. Extraction method was performed again by adding ethyl acetate and lastly extraction solution and phosphate buffer was mixed. The OD was determined at 254 nm by utilizing spectrophotometer (Berríos et al., 2004).

#### 2.6.2 Solubilization of potassium (K), zinc (Zn), and phosphate (P)

Potassium solubilization by the selected strains was quantitatively measured using Aleksandrov broth. The bacterial isolates were introduced into a 40 ml volume of Aleksandrov broth and subjected to incubation in a rotary shaker operating at 4.26 g for a duration of 5 days at a temperature of 30°C. As a control, broth was autoclaved. After incubation, pH of the broth was noted. Post incubation, the broth was centrifuged at a speed of 42,600 × g for 10 min. The supernatant was collected and then utilized for the purpose of measuring the concentration of soluble potassium using a flame photometer. Various concentrations (20, 30, and 40 mg L^−1^) of potassium chloride solution were prepared as standard solutions for the measurement of present K levels (Rajawat et al., 2016).

The potential of Zn solubilization by the isolated bacteria was quantitatively checked by following the protocol proposed by Fasim et al. (2002). Overnight grown bacterial cultures (2.5% inoculum) were inoculated in tris minimal salts medium (250 ml) supplied with 14 mM zinc oxide and non-supplemented medium was used as a control without inoculation. Bacterial strains were kept on orbital shaker to incubate for 10 days at 30°C. The pH of samples was measured every day. The medium was centrifuged and the supernatants were collected. The supernatants were acidified using 6 M HNO_3_ and the soluble ZN content was measured by atomic absorption spectrophotometry (AAS). The solubilized zinc content was determined by measuring the difference between the soluble zinc in the inoculation sample and the equivalent control and expressing it as grams of Zn ml^−1^ culture.

National botanical research institute's phosphate (NBRIP) medium was utilized to quantitatively measure the phosphate solubilizing ability of isolated bacteria (Nautiyal, 1999). The cultures were grown for 24 h and then 1 ml culture was inoculated into 50 ml NBRIP medium. The medium was put in an incubator at 17.04 × g for 6 days at 28±2°C in an orbital shaking incubator. The pH of the medium was recorded and uninoculated medium taken as a control. Post incubation, the cultures were subjected to centrifuge at 27,264 × g for 20 min and supernatants were obtained. Available phosphate in the culture supernatants were quantified by following the vanadomolybdophosphoric acid colorimetric method (Kumar et al., 2009).

#### 2.6.3 Determination of ammonia, 1-aminocyclopropane-1-carboxylate (ACC) deaminase, exopolysaccharides (EPS) production and nitrogen fixation capability

The evaluation of ammonia production, selected isolates were introduced into a solution of peptone water with a concentration of 1%. The mixture was then kept in an incubator at a 28°C for 24 h. Subsequently, a bacterial suspension was combined with 0.5 ml of Nessler's reagent. The conversion of a yellow to brown color signifies the generation of ammonia. The OD was measured at 450 nm, and the quantity of generated ammonia was calculated using ammonium sulfate standard curve. The standard curve covered a range of 0.1–1 μmol ml^−1^. The solubilized zinc content was determined by calculating the difference between the soluble zinc concentration in the inoculation sample and the equivalent control. This difference was then represented as a gram of Zn ml^−1^ culture. As a negative control, sterile peptone water was used. The potential of N_2_ fixation by bacterial isolates was checked using glucose nitrogen free mineral medium (GNFM medium). Transition of green to blue color of the medium during a 7-day incubation period at a temperature of 28°C, serves as an indicator of the isolate's capability for N_2_ fixation (Bashir et al., 2013).

Qualitative and quantitative assay to check 1-aminocyclopropane-1-carboxylate (ACC) deaminase production ability of isolated bacteria was performed by using the methods proposed by Dworkin and Foster (1958), Honma and Shimomura (1978), and Penrose and Glick (2003). The strains were inoculated on minimal salts media supplied with 3 mM ACC (as a source of nitrogen) to check the efficiency of ACC deaminase production. Inoculated media plates were kept in an incubator for 3 days at 28 ± 2°C and growth was monitored every day. Growth of bacterial strains on the plate indicates the production of ACC deaminase. The amount of α-ketobutyrate formed per mg of protein per h served as an indicator of the catalytic activity of enzyme ACC deaminase. A standard curve of α-ketobutyrate was prepared to standardize the mean value.

The exopolysaccharides (EPS) production efficiency of isolated strains was checked by inoculating bacterial cultures into TSB medium and kept in shaking incubator at 6.13 × g for 3 days at 30°C. Exopolysaccharides were separated from bacterial cultures by centrifuging them at 42,600 × g for 15 min at 4°C. Then the EPS were separated from the supernatant, which was collected in a volume/volume ratio of 3:1 using pre-chilled 95% ethanol. The EPS that had been precipitated was subjected to centrifugation for a duration of 20 min at a speed of 95,850 × g. To minimize potential inaccuracies, the sample was subjected to a drying process for a duration of 24 h at a temperature of 58°C within the confines of the centrifuge tube. Subsequently, the EPS weight was determined after the drying process (Verhoef et al., 2003).

#### 2.6.4 Biocontrol attributes

Siderophore formation by selected strains was checked using Chrome Azurol S (CAS) dye. Petri plates having bacterial inoculum, were kept in an incubator at 28 ± 2°C for 5–7 days and the formation of a yellow-orange halo zone surrounding the colonies confirms the production of siderophore (Schwyn and Neilands, 1987). King's B agar media supplied with glycine (4.4 g L^−1^) was used to check hydrogen cyanide (HCN) production efficiency. Whatman No. 1 filter paper soaked in 0.5% picric acid and 2% Na_2_CO_3_ and put on the upper lids of petri plates and wrapped using parafilm to make it airtight. Post incubation of at 28°C 3 days, yellow to orange-brown color change of Whatman No. 1 filter paper indicates the formation of HCN (Bakker and Schippers, 1987).

Synthesis of hydrolytic enzymes by the selected bacterial strains was performed according to Patel et al. (2023b). The isolated bacteria were subjected to spot inoculation on media plates and subsequently incubated for a duration of 48 h at a temperature of 30°C. This process was carried out using specific agar media for the detection of various enzymes, namely: (a) starch agar for amylase enzyme; (b) skimmed milk agar for protease enzyme; (c) chitin agar for chitinase enzyme; (d) pectinase screening agar medium for pectinase enzyme; and (e) carboxy methyl cellulose agar for cellulase enzyme. The presence of distinct hydrolytic enzymes was verified through the observation of a transparent zone around the colonies.

### 2.7 Abiotic stress tolerance ability

The strains that were chosen were cultivated in nutrient agar media in order to assess their capacity to withstand different abiotic stresses. These stresses included exposure to varying concentrations of sodium chloride (5%, 10%, 15%, and 20%), different pH levels (5, 7, and 9), growth efficiency at different concentrations of polyethylene glycol (PEG6000) for drought stress (10%, 20%, and 30%), and endurance at different temperatures (25°C, 37°C, 45°C, and 55°C). For each abiotic stress analysis, 100 ml of bacterial culture medium was made and put in an incubator at 30°C for 48 h. Post incubation, the OD was calculated at a wavelength of 600 nm to examine the growth of bacteria.

### 2.8 Antioxidant activity assay

About 1 ml of isolated bacteria's cell-free supernatant was combined with 3 ml of 0.1 mM 2-diphenyl-2-picryl hydrazyl hydrate (DPPH) and then allowed to incubate in dark condition for 30 min (Ashry et al., 2022). The OD was determined at a wavelength of 515 nm in triplicate. As a control, 3 ml DPPH solution and 1 ml ethanol was mixed. To measure DPPH scavenging activity, the following equation was used:


$$\begin{array}{l} \text{DPPH radicals scavenging activity }\left(\text{\%}\right)\\ =\;\frac{\left(\text{OD of the control}-\text{OD of the sample}\right)}{\text{OD of the sample}}\times 100 \end{array}$$

### 2.9 Statistical analysis

The experimental procedures were carried out in triplicate. Moreover, the mean value was derived as the final outcome. The statistical software SPSS version 25.0 was utilized to determine the statistical disparities between the treatments using Tukey's HSD (honestly significant difference) tests, with a significance level of *p* < 0.05 (Islam et al., 2016). The principal component analysis (PCA) and pairwise-correlation analysis were performed utilizing the “ggplot2 package” in the R.Studio software (version R 4.2.3) (Wickham, 2016).

## 3 Results

### 3.1 Jeevamrit and Beejamrit treatment alter the soil chemical properties and nutrient content

The soil analysis was conducted following JB treatment accompanied with salinity and drought stress. Under salinity condition, the pH and electrical conductivity increased while they declined in drought conditions (Table 2). The quantity of organic carbon increased following JB treatment, with the highest amount detected after salinity (4 dsm^−1^) treatment (Table 2). Plant nutritional elements (PNEs) and microbial load were both noticeably elevated in soils treated with JB (Table 2).

**Table 2:** The positive influence of JB treatment on soil physicochemical characteristics.

**Soil parameters**	**Pot experiment soil (before JB treatment)**	**Pot experiment soil (after JB treatment)**			
		**Salinity (4 dsm** ^−1^ **)**	**Salinity (6 dsm** ^−1^ **)**	**Drought (moderate)**	**Drought (severe)**
pH	7.3	8.1	8.7	6.9	6.6
Electrical conductivity (dS m^−1^)	0.54	4.0	6.0	0.51	0.49
Organic carbon (%)	0.72	1.1	0.94	0.98	0.91
Nitrogen (%)	0.32	1.3	1.1	1.2	1.2
Phosphorus (kg/ha)	43	67	54	71	59
Potassium (kg/ha)	279	312	298	306	295
Calcium (kg/ha)	163	188	179	185	181
Zinc (ppm)	27.2	29.8	28.4	29.2	28.1
Manganese (ppm)	29.1	30.7	29.6	30.9	29.8
Copper (ppm)	11.3	13.1	12.6	12.7	12.2
Iron (ppm)	36.8	38.9	37.4	39.2	37.8
Microbial load (cfu g^−1^ soil)	34.8	60.4	49.8	57.3	46.5

### 3.2 Jeevamrit and Beejamrit treatment can augment spinach growth under salt and drought stress

**Figure 1:**
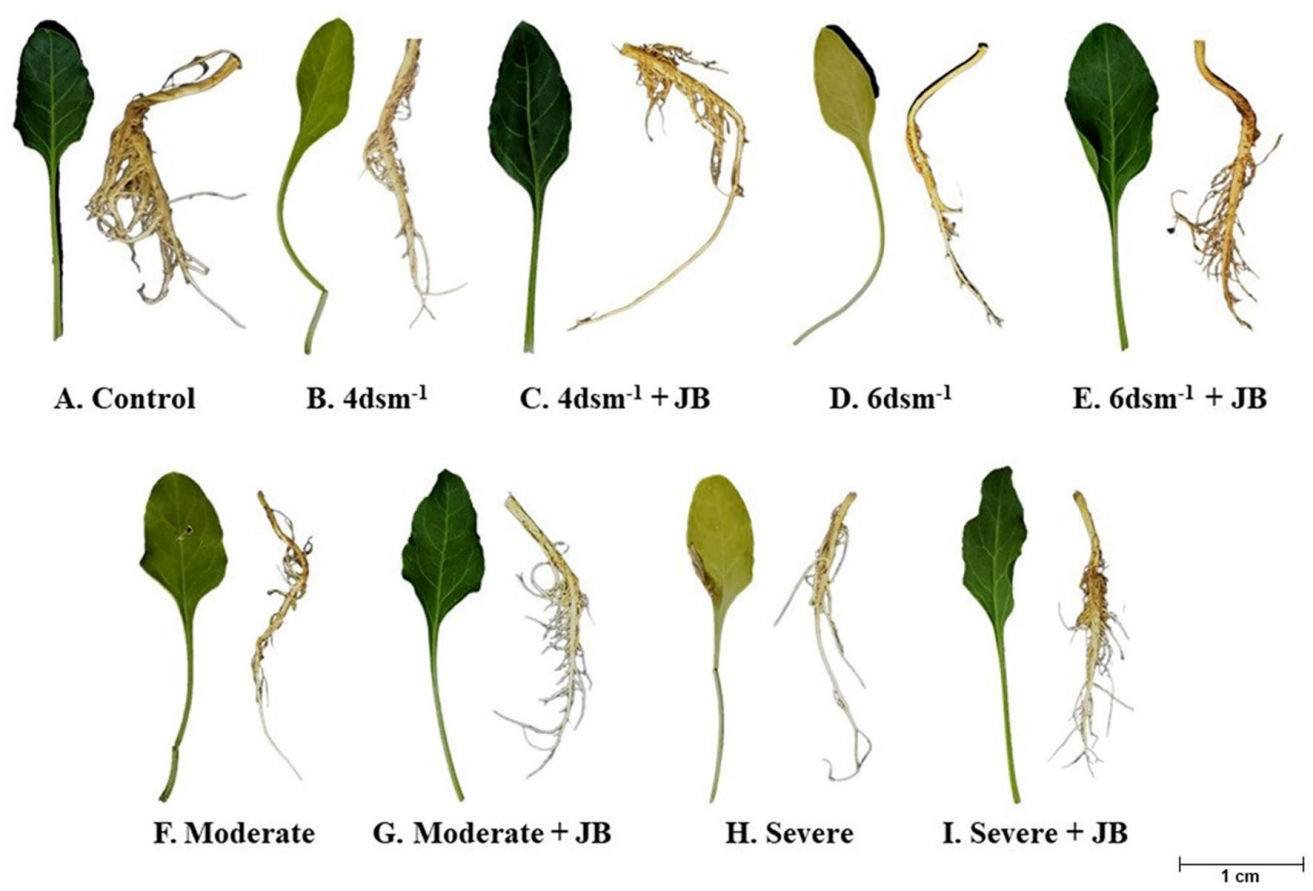
Growth promotion of spinach plant following JB treatment under salt (4 and 6 dsm^−1^) and drought (Moderate and Severe) conditions.

**Figure 2:**
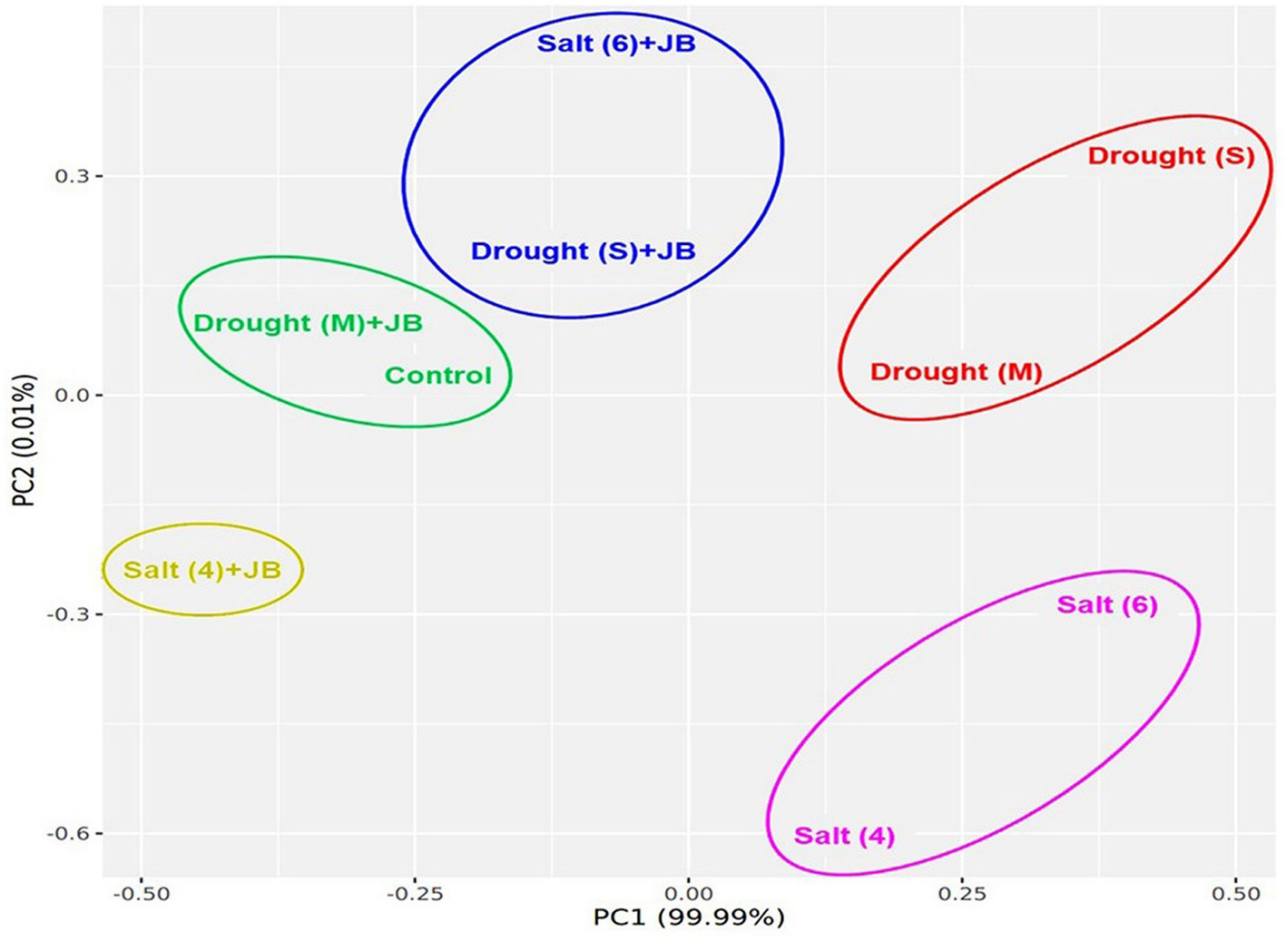
JB treatment positively regulate the spinach growth under salt and drought stress. Data from Table 3 was utilized to run PCA for pairwise analysis. ggplot2 package of the R programming software were used for PCA analysis.

**Figure 3:**
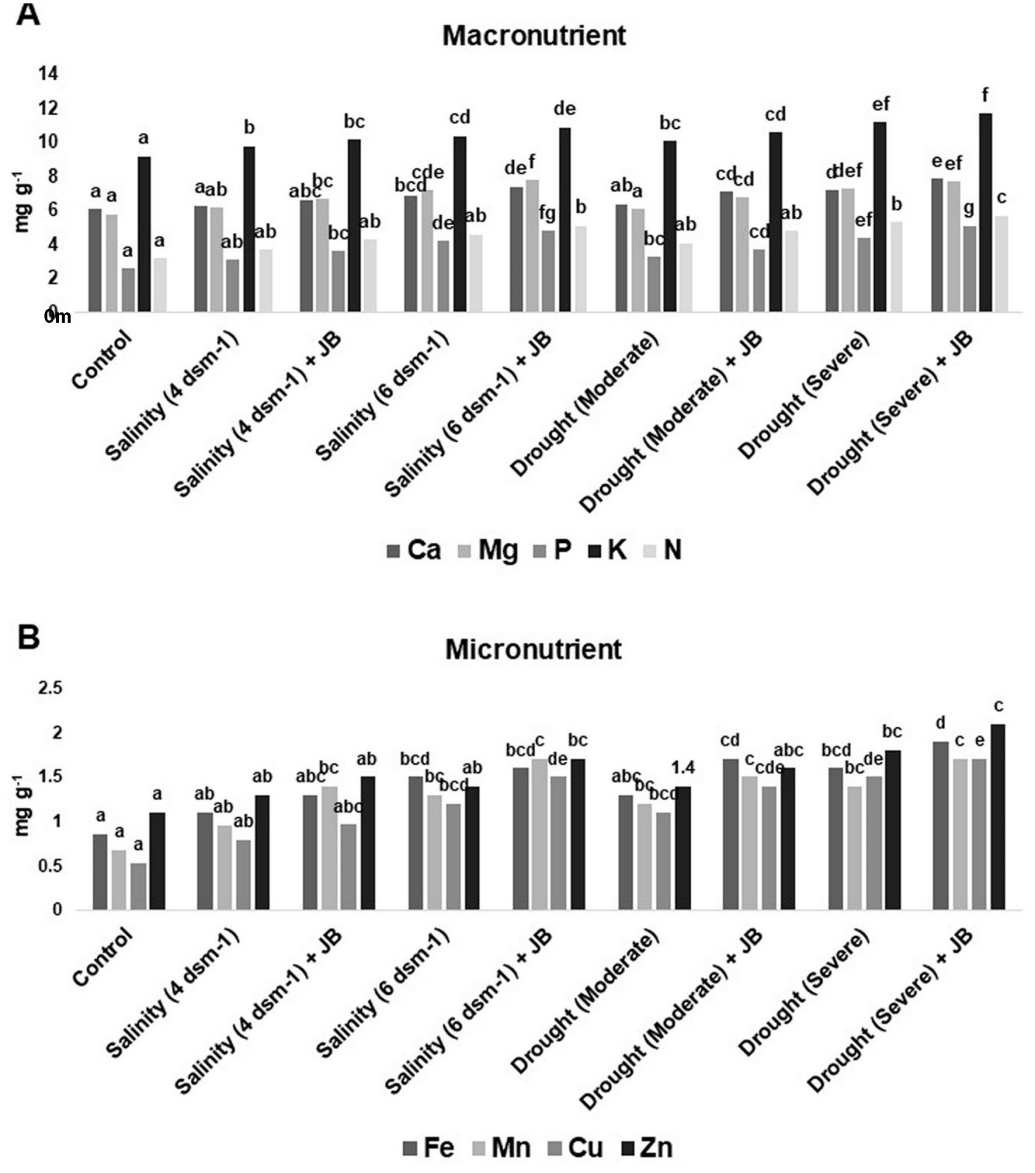
Effect of strain JB treatment on **(A)** macronutrient content and **(B)** micronutrient content. The data represents the total amount (mg g^−1^) of minerals in each of three sets of 8–10 samples. In each frame, distinct letters were used to represent a statistically significant distinction (*p* ≤ 0.05) between the various treatments. There was a minimum of two further replications of the experiment.

The JB treatment exhibited a significant improvement in spinach plant growth (*p* ≤ 0.05). The treatment combination of salinity (4 ds m^−1^) + JB displayed the highest values for plant biomass, plant length, leaf area, germination (%), and vigor index. This was followed by the treatment combination of moderate drought + JB (Table 3 and Figure 1). Treatment of JB significantly (*p* ≤ 0.05) recovered the spinach plant growth under both the conditions—drought (severe) + JB and salinity (6 ds m^−1^) + JB (Table 3). Principal component analysis (PCA) with the Supplementary Table S1 data was carried out to further establish the significant differential response of the spinach plant growth to JB treatment (Figure 2). The results of PCA (Supplementary Table S2) indicated that six distinct clusters were formed, where the drought (moderate) + JB treatment data were clustered with the non-treated control plants (Figure 2). Consequently, the use of JB treatment serves to lessen the detrimental effects of salt exposure and water deficiency situations on spinach plants. Additionally, spinach plant's concentrations of both micronutrients (Cu, Mn, Zn, and Fe) and macronutrients (K, Ca, N, P, and Mg) greatly increased following JB treatment (Figures 3A, B).

**Table 3:** Effect of JB treatment on spinach growth under salt and drought stress.

**Treatments**	**Germination (%)**	**Leaf width (cm)**	**Leaf length (cm)**	**Leaf area (cm^2^)**	**Shoot height (cm)**	**Root length (cm)**	**Shoot fresh weight (g)**	**Shoot dry weight (g)**	**Root fresh weight (g)**	**Root dry weight (g)**	**Vigor index**
Control	70f	6.9e	11.39cd	78.59f	9.31c	12.5c	2.14d	1.56e	1.14e	0.83d	2324g
Salt (4)	50d	4.3b	10.23a	43.98b	8.37a	10.4a	1.35b	0.98c	0.72c	0.41b	1450d
Salt (4) + JB	80g	7.4f	11.5cd	85.1i	9.4cd	15.1e	2.58e	1.88f	1.21f	0.96e	2880i
Salt (6)	30b	3.7a	10.06a	37.22a	8.24a	10.7a	1.03a	0.75a	0.55a	0.21a	870b
Salt (6) + JB	60e	7.1ef	11.28bc	80.08g	9.22c	11.6b	1.75c	1.27d	0.93d	0.64c	1926e
Drought (M)	40c	5.1c	10.12a	51.61d	8.28a	10.5a	1.2b	0.87b	0.64b	0.39b	1156c
Drought (M) + JB	70f	7.2f	11.77d	84.74h	9.63d	14.8d	2.05d	1.49e	1.09e	0.8d	2534h
Drought (S)	20a	4.1b	10.94b	44.85c	8.96b	10.7a	0.99a	0.72a	0.53a	0.15a	612a
Drought (S) + JB	60e	6.4d	11.6cd	74.24e	9.5cd	11.8b	1.84c	1.34d	0.98d	0.67c	1974f

Inside each frame, different letters are utilized to show a statistically significant (p ≤ 0.05) difference between treatments. At least two further repetitions of the experiment were performed.

### 3.3 Effect of Jeevamrit and Beejamrit treatment on antioxidant enzyme activity

**Figure 4:**
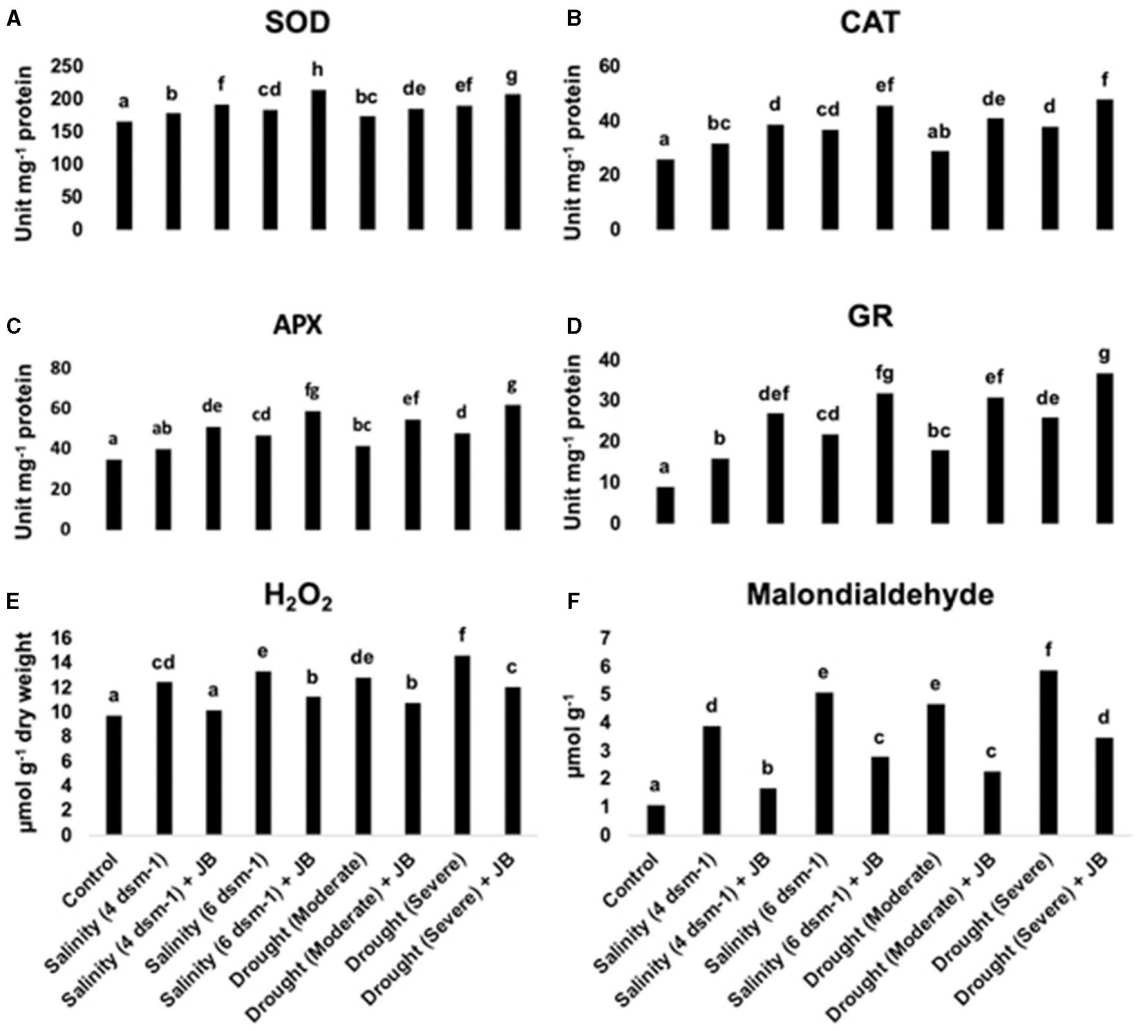
**(A–F)** The antioxidant enzyme activity and reactive oxygen species (ROS) levels in spinach plants are positively regulated by JB treatment. In each frame, distinct letters were used to represent a statistically significant distinction (*p* ≤ 0.05) between the various treatments. There were at least two further repetitions of the experiment. Where, SOD, superoxide dismutase; CAT, catalase; APX, ascorbate peroxidase; GR, glutathione reductase; H_2_O_2_, hydrogen peroxide.

Under drought and salinity stress conditions, JB treatment demonstrated a notable augmentation in the activity of the enzymes glutathione reductase (GR), catalase (CAT), superoxide dismutase (SOD), and ascorbate peroxidase (APX), as depicted in Figures 4A–D. In contrast, the JB application showed a decline in the levels of malondialdehyde (MDA) and hydrogen peroxide (H_2_O_2_), as depicted in Figures 4E, F. Application of JB can thereby lessen the adverse impact that salt and drought stress exert on spinach plants.

### 3.4 The quantity of total free amino acids, chlorophyll, and total soluble sugars increased significantly following Jeevamrit and Beejamrit treatment

**Figure 5:**
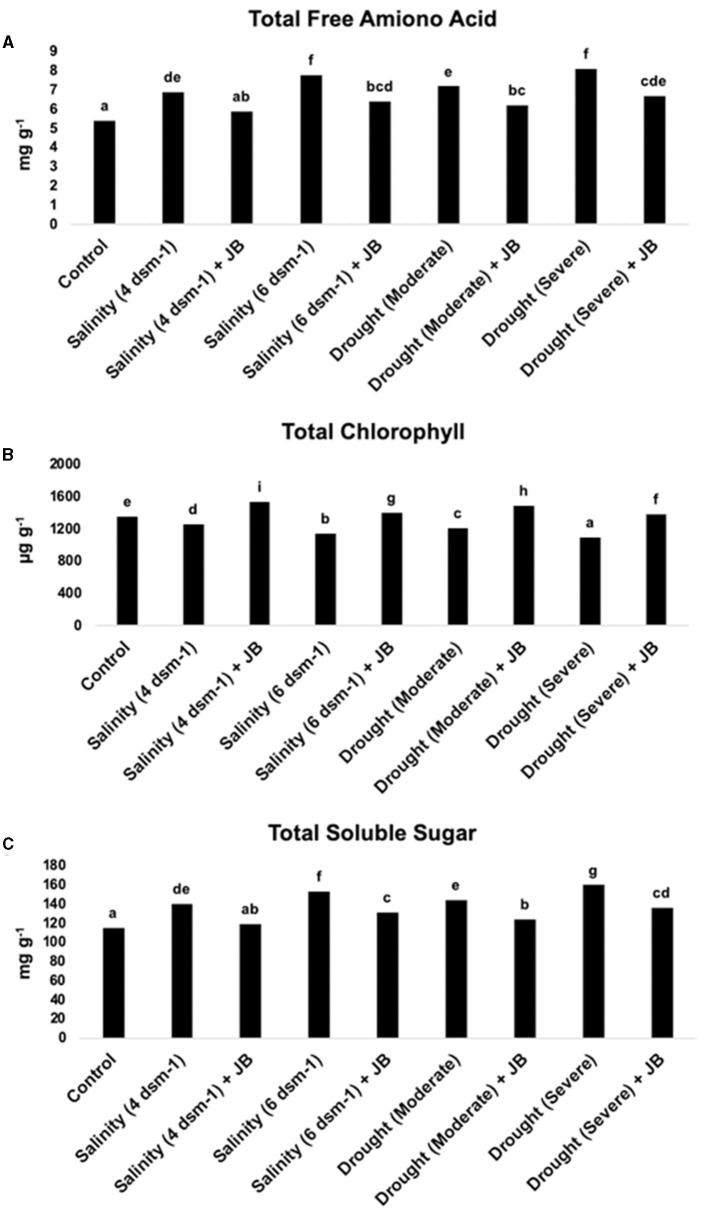
**(A–C)** The effect of JB treatment on total soluble sugars (TSS), total chlorophyll, and total free amino acids. In each frame, distinct letters were used to represent a statistically significant distinction (*p* ≤ 0.05) between the various treatments. The experiment was repeated at least two more times.

The results noticeably exhibit that the application of JB treatment had a substantial effect on the overall levels of total chlorophyll, total free amino acid, and total soluble sugar in spinach (Figure 5).

### 3.5 Jeevamrit and Beejamrit treatment demonstrates a significant reduction in glycine betaine and proline amount in spinach plants under salinity and drought stress

**Figure 6:**
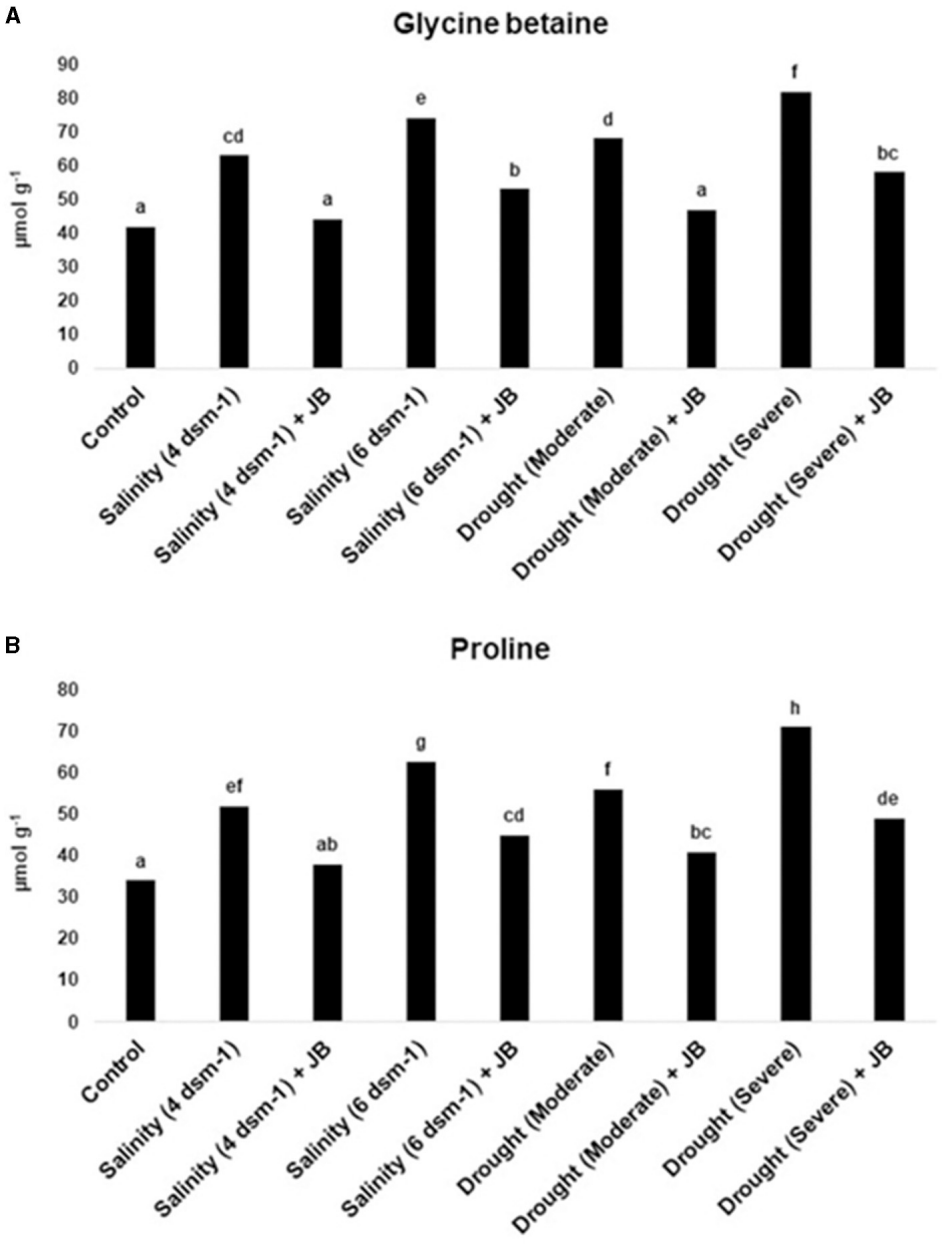
**(A, B)** Lower levels of glycine betaine and proline were found in spinach plants that had been exposed to JB, demonstrating the JB's capacity to induce defense against abiotic stresses. In each frame, distinct letters were used to represent a statistically significant distinction (*p* ≤ 0.05) between the various treatments. The experiment was repeated at least twice.

A noteworthy decrease in proline and glycine betaine amount was reported in spinach subsequent to treatment with JB (Figure 6).

### 3.6 Prevention of stress-induced cell death and the increase in relative water content in spinach plants mediated by Jeevamrit and Beejamrit

**Figure 7:**
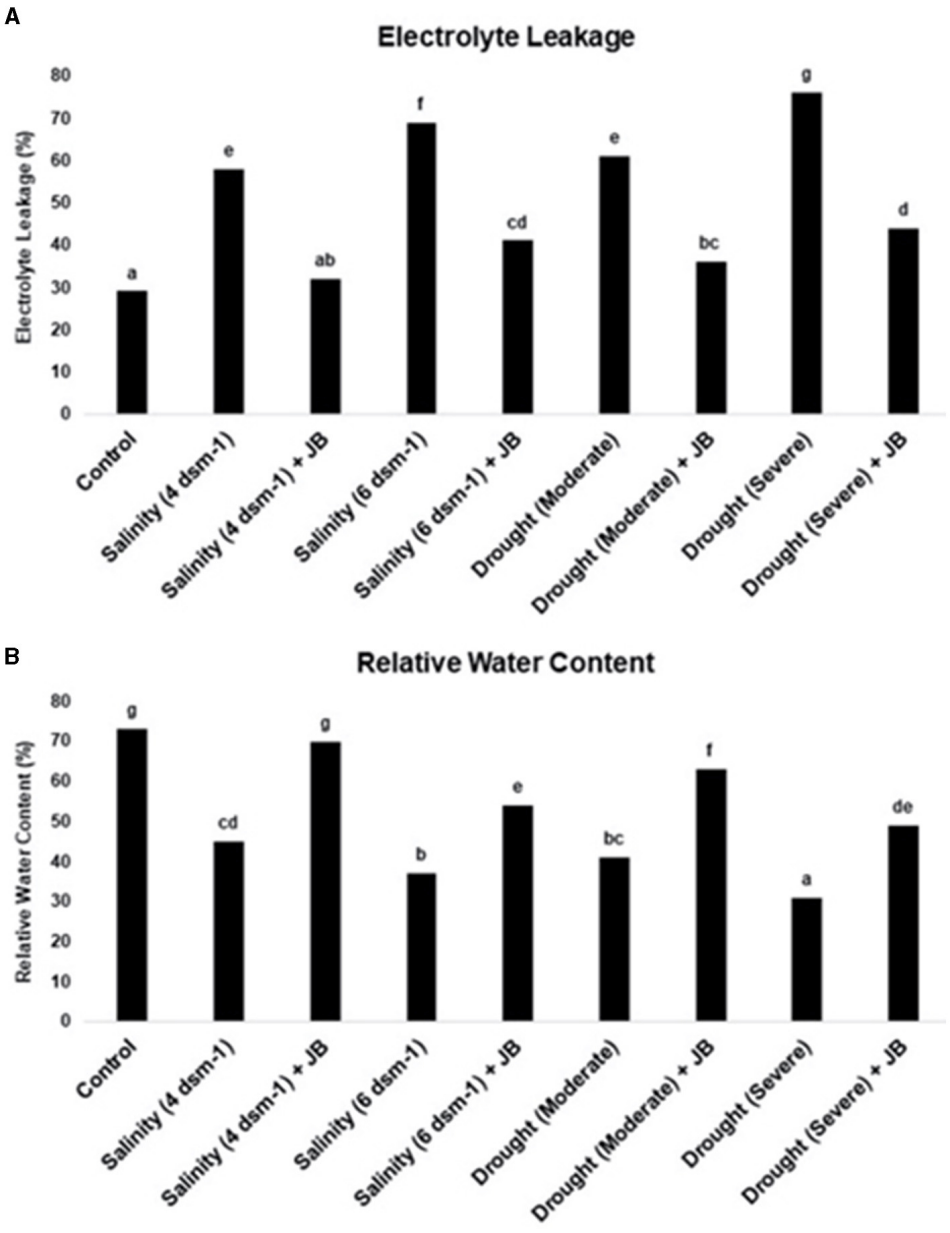
**(A, B)** The influence of JB treatment on the rate of electrolyte leakage and relative water content. In each frame, distinct letters were used to represent a statistically significant distinction (*p* ≤ 0.05) between the various treatments. The experiment was repeated at least twice.

The results obtained from the experiment on electrolyte leakage indicate that the use of JB treatment leads to a considerable decrease in cell mortality in the treated plants. Additionally, it was observed that the relative water content (RWC) of spinach plants increased as a result of the JB treatment, as depicted in Figure 7. The drought (severe) treatment resulted in the highest level of cell death, whereas the salinity treatment at 6 ds m^−1^ exhibited the lowest RWC (Figure 7). The data unequivocally demonstrates the significant mitigation of salinity and severe drought's detrimental impacts with JB treatment.

### 3.7 Isolation of bacterial strains, biochemical and molecular characterization

Analyses were conducted on the five bacterial isolates isolated from the JB formulation to see how they might promote crop growth and help the plants endure abiotic stresses. Supplementary Table S3 exhibits the biochemical features of the five bacterial isolates. *Enterococcus faecalis, Bacillus tequilensis*, and *Bacillus tropicus* are gram positive, while *Shigella sonnei*, and *Morganella morganii* are gram negative (Supplementary Table S3). Except for *E. faecalis*, the other four isolates are rod-shaped and both the *Bacillu*s species as well as *M. morganii* are spore forming, motile bacteria (Supplementary Table S3). *Shigella sonnei* and *M. morganii* can produce indole, while *B. tequilensis* and *B. tropicus* can consume citrate as a C (Carbon) source (Supplementary Table S3). Three of the five isolated strains can produce the extracellular proteolytic gelatinases and catalases. Furthermore, based on the affirmative VP test outcome, it has been observed that *E. faecalis, B. tequilensis*, and *B. tropicus* possess the capability to utilize the metabolic pathway of butylene glycol in order to produce acetoin. The bacterial isolates have the ability to undergo fermentation of various sugar sources, such as sucrose, lactose, dextrose, and fructose. This has been verified using the sugar fermentation test, as indicated in Supplementary Table S3.

**Figure 8:**
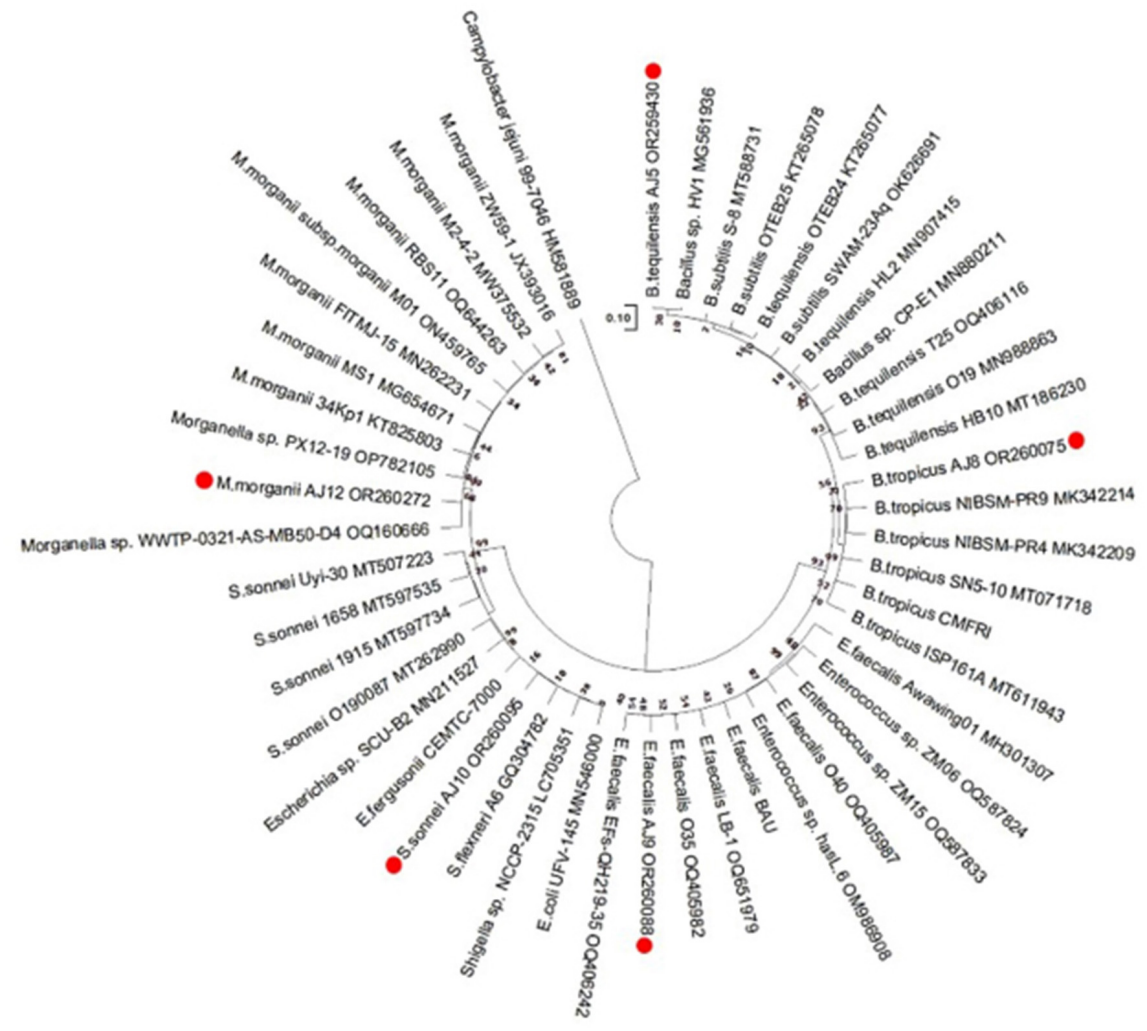
The results of the evolutionary analysis, which was carried out using the Tamura-Nei model and the Maximum Likelihood method, are shown in the phylogenetic tree. The tree with the maximum log likelihood value (−53,781.84) is displayed. The initial trees for the heuristic search were obtained using the automated application of the Maximum Parsimony approach. With branch lengths expressed as the number of substitutions per site, the tree is scaled. The present study encompassed a total of 48 nucleotide sequences. The completed dataset consisted of a total of 34,376 locations. Evolutionary analyses were performed utilizing the software MEGA11.

By sequencing the 16S rRNA gene, these five bacterial isolates were molecularly identified. Figure 8 illustrates the maximum-likelihood tree that was generated using the sequences of the five bacteria mentioned, along with the associated reference sequences obtained from the NCBI database. The details about the NCBI data and accession numbers of these five isolates are shown in Supplementary Table S4. The bacterium *Campylobacter jejuni* was used as an outgroup representative. A phylogenetic tree was generated in order to evaluate the point at which these bacterial isolates diverge from other genera of the pertinent bacterial ancestors (Figure 8). The Supplementary Table S5 displays the “Patristic distances,” which represent the estimations of evolutionary divergence between the sequences.

### 3.8 Plant growth promoting attributes of the isolated bacterial strains

All five bacterial strains produced indole-3-acetic acid (IAA), gibberellic acid (GA_3_), ammonia, 1-aminocyclopropane-1-carboxylate (ACC) deaminase, and exopolysaccharides (EPS; Table 4). The highest IAA and GA_3_ concentrations were produced by the isolate *B. tropicus* followed by the isolate *B. tequilensis* (Table 4). *Bacillus tequilensis* produced 5.8 μmol ml^−1^ ammonia, while *B. tropicus* produced 15.7 μmol h^−1^ mg^−1^ of ACC deaminase (Table 4). With the exception of *S. sonnei*, the other four bacteria were able to fix atmospheric nitrogen. All five isolates solubilized inorganic phosphate with the highest value (43.6 μg ml^−1^) being found in *M. morganii*, although it could not solubilize zinc (Zn; Table 4). All other isolates were able to solubilize Zn and potassium except from *B. tropicus*. Moreover, these isolates contain several biocontrol attributes. Siderophore, hydrogen cyanide (HCN), Protease, Amylase, Chitinase, and Cellulase—were produced by these bacterial isolates (Table 4).

**Table 4:** Beneficial properties of the bacterial isolates that encourage the spinach plant growth.

**PGP traits**	**Isolates**				
	***Enterococcus faecalis*** **AJ9**	***Bacillus tequilensis*** **AJ5**	***Shigella sonnei*** **AJ10**	***Morganella morganii*** **AJ12**	***Bacillus tropicus*** **AJ8**
Indole-3-acetic acid (μg ml^−1^) production	98.4	88.3	46.9	76.9	114.8
Gibberellic acid (μg ml^−1^) production	53.8	64.9	29.3	36.4	71.1
Ammonia (μmol ml^−1^) production	4.9	5.8	3.5	4.2	5.1
1-aminocyclopropane-1-carboxylate (ACC) deaminase (μmol h^−1^ mg^−1^ of α-ketobutyrate protein) production	11.5	10.7	8.2	13.9	15.7
Exopolysaccharides (g L^−1^) production	24.6	18.5	20.1	17.9	29.4
Nitrogen fixation	+	++	–	++	+++
Phosphorus solubilization (μg ml^−1^)	41.3	26.5	38.1	43.6	31.9
Potassium solubilization (μg ml^−1^)	29.3	35.7	21.6	18.2	–
Zinc solubilization (μg ml^−1^)	24.6	31.7	45.2	-	37.9
**Biocontrol attributes**					
Siderophore production	–	+	++	+++	++
Hydrogen cyanide production	+++	+++	++	+	+
Protease production	+++	+	–	++	+
Amylase production	–	++	+++	+	++
Chitinase production	++	–	++	–	+
Cellulase production	++	+++	+	+++	++

(+) = positive test; (–) = negative test; (++) = good; (+++) = excellent.

### 3.9 Abiotic stress tolerance of the bacterial isolates

All five bacterial isolates have shown varying degree of tolerance to different abiotic stress parameters (Table 5). *Bacillus tropicus* AJ8 and *E. faecalis* AJ9 have demonstrated the ability to tolerate sodium chloride (NaCl) concentrations of up to 20%, while the other three bacteria have also demonstrated tolerance to high salt concentrations (Table 5). With the exception of *M. morganii* AJ12, which can only grow at pH 7.0, the other four bacteria have shown resilience to pH fluctuations (Table 5). All five bacteria can tolerate temperatures as high as 45°C, however, *E. faecalis* AJ9 and *B. tropicus* AJ8 cannot survive temperatures as high as 55°C (Table 5). Moreover, all five bacterial isolates have shown tolerance to different level of PEG concentrations (Table 5). The isolates are therefore very likely to help plants develop abiotic tolerance.

**Table 5:** Bacterial isolates showing substantial tolerance against various abiotic stresses.

**Isolates**	**Abiotic stress tolerance parameters**													
	**Salt (NaCl—%)**				**Temperature (** ^o^ **C)**				**pH**			**Drought (PEG–%)**		
	**5%**	**10%**	**15%**	**20%**	**25** ^o^ **C**	**35** ^o^ **C**	**45** ^o^ **C**	**55** ^o^ **C**	**5**	**7**	**9**	**10%**	**20%**	**30%**
*Enterococcus faecalis* AJ9	√	√	√	√	√	√	√	×	×	√	√	√	√	√
*Bacillus tequilensis* AJ5	√	√	√	×	√	√	√	√	×	√	√	√	√	√
*Shigella sonnei* AJ10	√	√	×	×	√	√	√	√	√	√	×	√	√	×
*Morganella morganii* AJ12	√	√	√	×	√	√	√	√	×	√	×	√	√	×
*Bacillus tropicus* AJ8	√	√	√	√	√	√	√	×	×	√	√	√	√	√

### 3.10 DPPH radical scavenging activity

**Figure 9:**
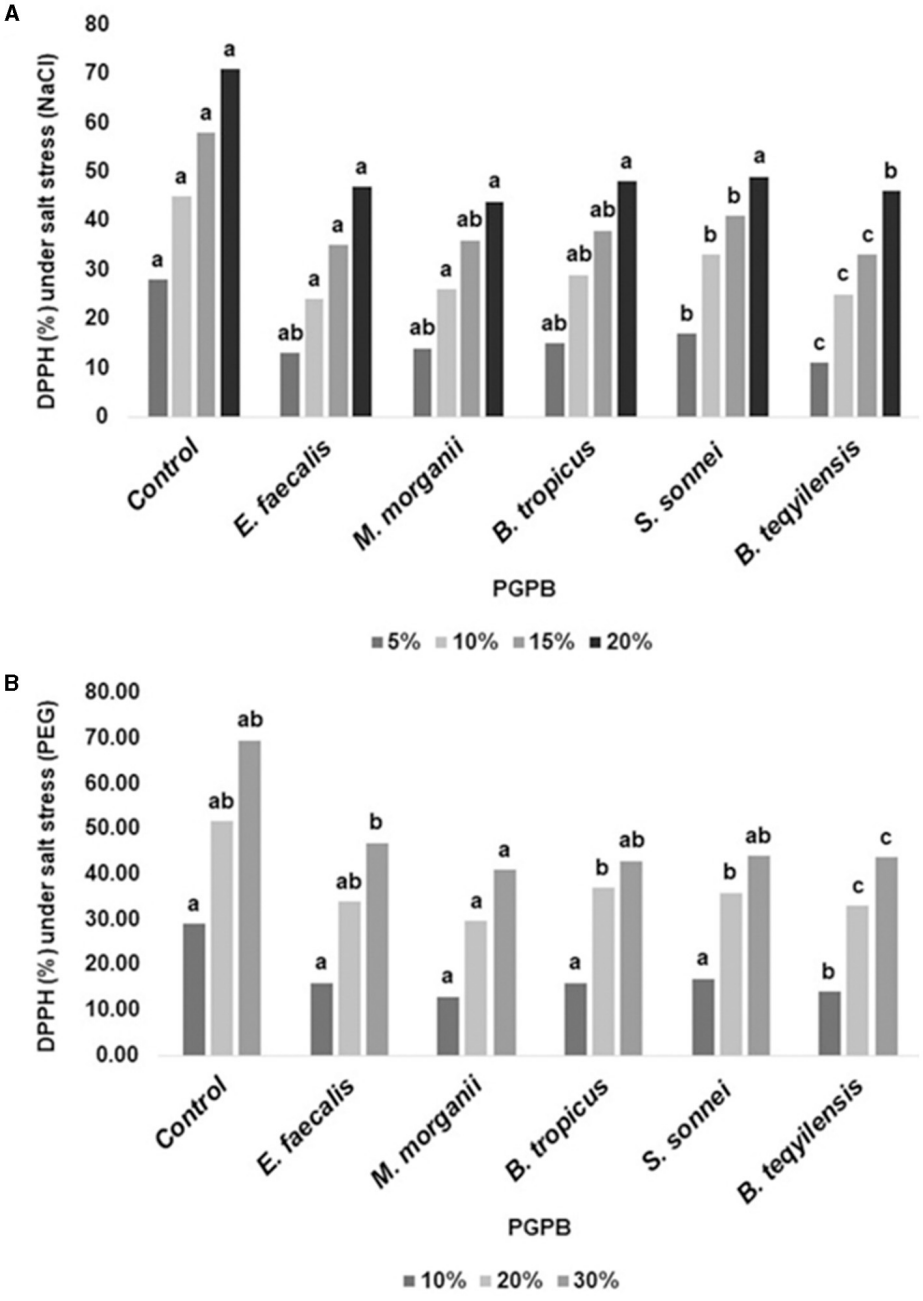
**(A, B)** The impact of the bacterial isolates on activity of DPPH radical scavenging. The experiment was repeated at least twice.

The detrimental consequences of the activity of DPPH radical scavenging were greatly mitigated by all five bacterial isolates, as shown in Figure 9.

**Figure 10:**
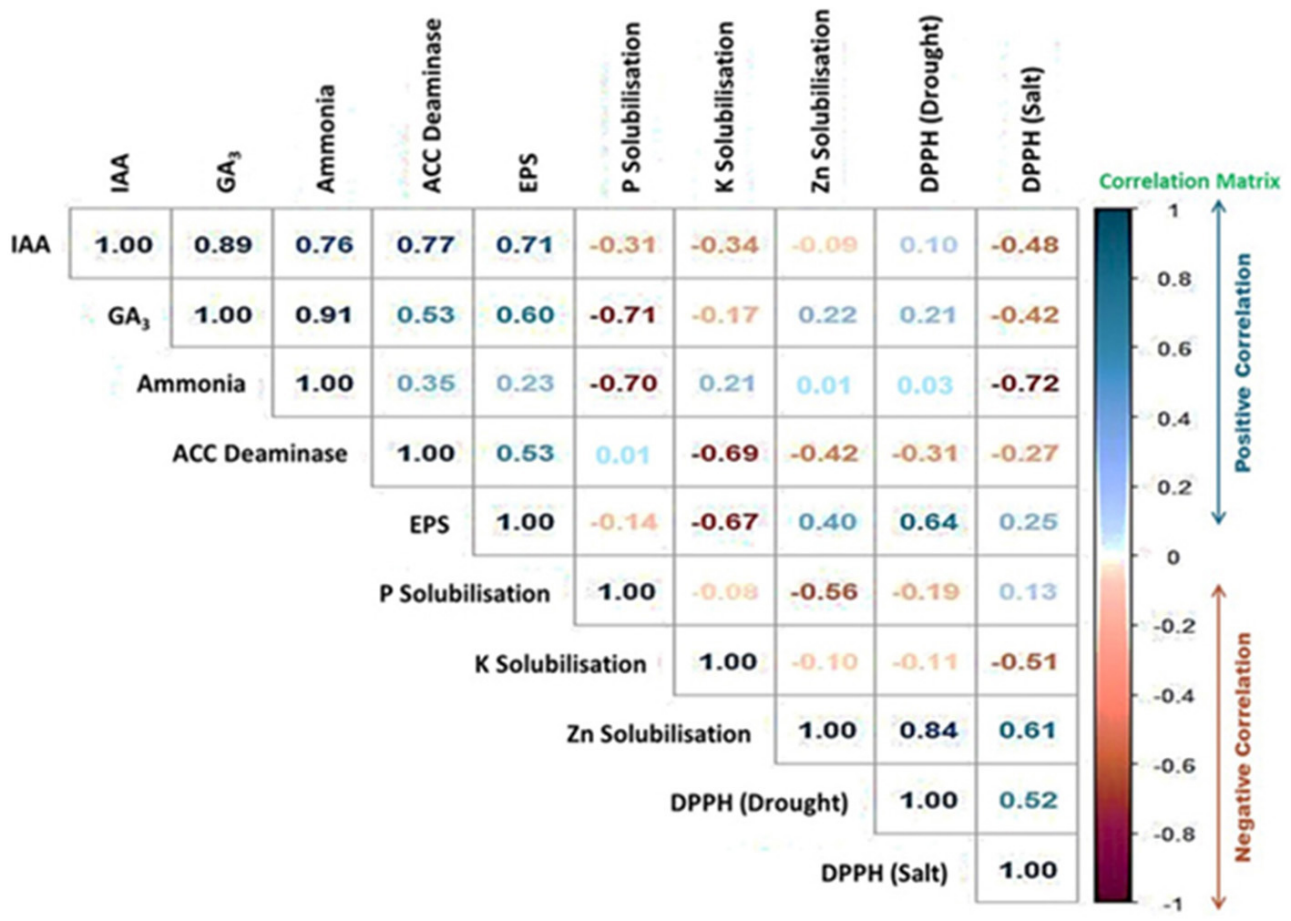
The correlation plot showing the positive (Blue) and negative (Red) correlation between the PGP traits of the PGPB isolates and DPPH radical scavenging activity. The “Legend” displays the values of the Pearson Correlation Coefficient (PCC).

Additionally, we found an intriguing relationship amid the DPPH radical scavenging activity and the PGP characteristics of the PGPB isolates. Figure 10 and Supplementary Table S6 demonstrate the outcome of the pairwise correlation analysis, which was executed using the data provided in Supplementary Table S7. Figure 10 shows a significant positive correlation between the PGP features of IAA production, GA_3_ production, EPS production, ammonia synthesis, and ACC Deaminase activity. Interestingly, EPS production by the PGPB isolates has demonstrated a strong correlation with DPPH scavenging activity under drought conditions (correlation matrix = 0.64). In contrast, Zn solubilization by the PGPB isolates was strongly correlated with DPPH scavenging activity under both drought and salt conditions (correlation matrix = 0.84 and 0.61, respectively; Figure 10).

## 4 Discussion

Climate change results from various human-induced activities that lead to global warming and other inescapable elements. Abiotic pressures such as salinity and drought are spurred on by unpredictable shifts in the climate, which eventually reduce agricultural output and damage natural resources (Shahzad et al., 2018). The bulk of these abiotic stress events, that vegetation experiences at different stages of their life cycle, cause crops to establish a variety of defensive responses, which can have the unintended and harmful effect of altering their physiological functions (Manzoor et al., 2022). Organic agriculture has been utilized for decades as an ecologically sound way to boost crop yields while protecting the environment. Additionally, it defends against a variety of frequently occurring biotic and abiotic stressors (Sleighter et al., 2023).

Therefore, this study examines the efficacy and functioning of the traditional fertilizers Jeevamrit and Beejamrit to overcome abiotic stresses of spinach plants. A wide range of biochemical and physiological assays and assessments have been employed to evaluate the effects of abiotic stressors, which include excessive salt and water deficit conditions. The use of these conventional fertilizers was found to largely (or at minimum in part) offset the negative/inhibitory stimuli of salinity and drought on spinach plants.

Application of JB significantly increased the content of the plant nutrient elements (PNEs) in the spinach plants. It is well-established that JB treatments boost soil's naturally occurring advantageous features and the plant's macro- and micronutrients (Mukherjee et al., 2022; Saharan et al., 2023). Earlier findings have also shown that JB application ensures the—(i) better regulation of the soil pH, (ii) expand the diversity of the rhizomicrobiome in the soil, (iii) application of fertilizer regularly and effectively since the JB formulation can be prepared in only 4–5 days, (iv) versatility since JB treatment works perfectly for a wide variety of crops, (v) fertigation with irrigation water, and (vi) greater yields while decreasing chemical fertilizer expenditures (Aulakh et al., 2013; Kumari et al., 2022; Saharan et al., 2023).

**Figure 11:**
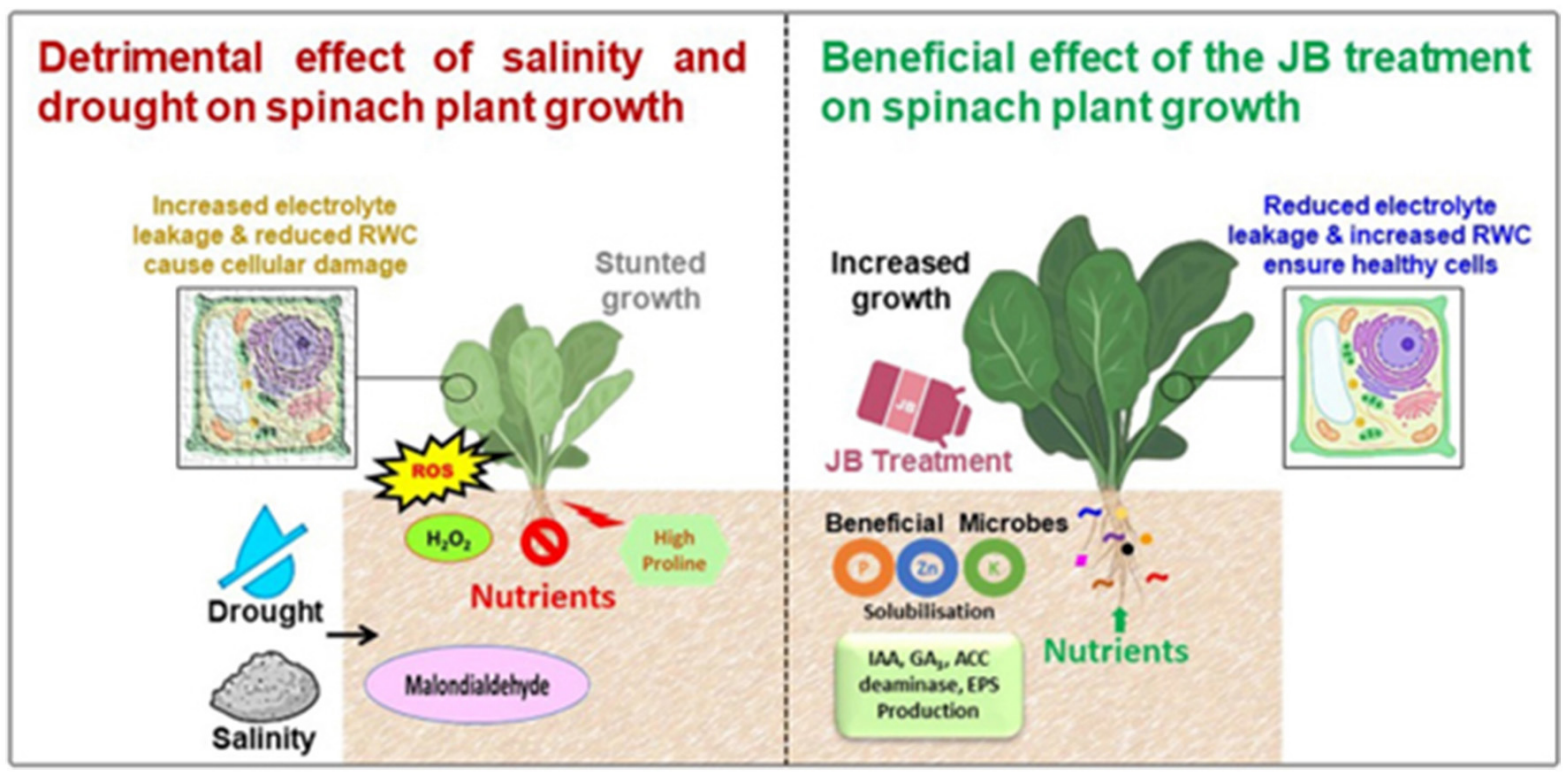
Schematic diagram showing the possible mechanisms of the JB-mediated mitigation of the salt- and water scarcity-induced stress in spinach plants. Drought along with the saline conditions cause the reduced soil fertility and thus inhibit/reduce the nutrient uptake by the plants. These leads to the hormonal imbalance and reduced photosynthesis in the drought stressed plants. Higher proline and glycine betaine content induce the oxidative stress. All these events lead to the growth suppression in the plants. JB treatment significantly increase the soil fertility and nutrient content in the spinach plants. JB treatment also induce the activation of antioxidant enzymes and reduce reactive oxygen species (ROS) generation. Moreover, JB treatment increase the beneficial microbes. In the plant rhizosphere and these microbes produce indole-3-acetic acid (IAA), gibberellic acid (GA_3_), 1-aminocyclopropane-1-carboxylate (ACC) deaminase, exopolysaccharides (EPS), etc. Finally, JB reduce the proline and glycine betaine content as well as ensure higher relative water content (RWC) that ultimately augment the spinach plant growth.

Natural farming relies primarily on the heightened biological processes occurring inside the soil (Prasad, 2023). Therefore, to comprehend the mechanisms for the effectiveness of the tested traditional fertilizers, five bacterial strains were isolated and characterized from these traditional fertilizers. All five bacterial strains were thoroughly characterized and found to have various plant growth-promoting activities. These results are consistent with the suggestion that the reason for the effectiveness of the traditional fertilizers was the presence of these (and possibly other) plant growth-promoting bacterial strains present within these traditional fertilizers. Figure 11 illustrates a summary of the numerous benefits of employing JB to alleviate the detrimental impact of salinity and water deficit revealed during the current study.

Our findings clearly demonstrate a detailed scientific rational to clarify the effectiveness of environmentally friendly zero budget natural farming. This study makes it clear that instead of trying to replace problematic and expensive (in the developing world) agricultural chemicals with individual rhizobacteria (Glick, 2020) or even with consortia of PGPB (Santoyo et al., 2021), it may be more expeditious (and much less costly) to depend upon zero budget natural farming which has previously been shown to increase the number of plant helpful bacteria (Maduka and Udensi, 2019). The presence of a strong rhizosphere microbiome is crucial for the proper functioning of ecosystem processes such as carbon and water cycling, nitrogen fixation, agricultural productivity, and carbon sequestration (Adl, 2016). The rhizosphere, a complex environment surrounding plant roots, is influenced by various interrelated factors, including substrate type, moisture levels, microbial composition, and plant physiology. These factors interact in a dynamic manner, giving rise to a range of interdependent processes within the rhizosphere (Ryan et al., 2009). Therefore, zero budget natural farming could be a competent and resourceful approach to engineer the crop rhizosphere.

Interestingly, we have found strong positive correlation between EPS production and drought tolerance in spinach plants (Figure 10). Under stressful circumstances, microbial EPS generation is a type of physiological adaptation that helps bacteria survive (Ashry et al., 2022). As a result, for assessing how well bacteria tolerate drought, the capacity of bacterial cells to produce EPS is utilized (Sandhya et al., 2009). Moreover, Zinc solubilization has shown positive correlation with both the salt and drought stress mitigation (Figure 10). By boosting growth-related biological functioning, which includes chlorophyll content, carotenoid, and antioxidant enzymes catalase and peroxidase, zinc-solubilizing bacteria protect crops from salinity-related damages (Prajapati et al., 2022; Srithaworn et al., 2023). Therefore, JB treatment has shown a number of favorable traits that may be highly useful in minimizing the harmful impacts of salt drought stress in an environmentally conscious and sustainable way.

## 5 Conclusion

Environmental stresses brought on by the unpredictably shifting climate have a disastrous influence on farming practices. Utilizing sustainable agricultural strategies emerges as the most efficacious approach to meet the escalating food demand while concurrently preserving the ecological wellbeing. The findings of this study bring new insights and futuristic approaches for sustainable agricultural productivity to cope with unfavorably changing climatic situations. Moreover, the results demonstrate unequivocally that Jeevamrit and Beejamrit, two traditional biofertilizers, include a variety of PGPB that can safeguard plants from abiotic challenges such as high salinity levels and water scarcity. These traditional fertilizers can also improve soil fertility by increasing plant nutritional components, which stimulate plant growth without the need for agrochemicals. Therefore, to protect the environment, eco-friendly agricultural approaches that promote sustainable agriculture should be explored and implemented comprehensively.

## Data availability statement

The original contributions presented in the study are included in the article/Supplementary material, further inquiries can be directed to the corresponding authors.

## Author contributions

MP: Formal analysis, Investigation, Methodology, Writing—original draft, Writing—review & editing. SI: Data curation, Investigation, Methodology, Writing—original draft, Writing—review & editing. BG: Formal analysis, Supervision, Validation, Visualization, Writing—review & editing. NC: Formal analysis, Investigation, Methodology, Software, Writing—review & editing. VY: Investigation, Methodology, Project administration, Writing—original draft, Writing—review & editing. SB: Conceptualization, Resources, Supervision, Visualization, Writing—review & editing. DS: Data curation, Formal analysis, Funding acquisition, Software, Validation, Writing—review & editing. AP: Conceptualization, Funding acquisition, Project administration, Supervision, Visualization, Writing—original draft, Writing—review & editing.
